# Supplementary material for: Are carbon emissions trading and green financial instruments synergistic? -Comprehensive quantitative research based on content analysis
Source: PLoS One. 2024 Mar 7;19(3):e0298601. doi: 10.1371/journal.pone.0298601 (PMC10919601; doi:10.1371/journal.pone.0298601)
Supplement: S1 Data — (ZIP) [file pone.0298601.s001.zip › Data/地级市Z_F工作报告.zip!/║╙─╧/╨┬╧τ╩╨/└┤╫╘╣1⁄2╓┌║┼í╛┬φ┐╦╩2╛▌═°í┐_2013.docx]

各位代表：

　　现在，我代表市人民政府，向大会作工作报告，请予审议，并请各位政协委员和特邀列席人员提出意见。

　　一、2012年工作回顾

　　2012年，在上级和市委正确领导下，在市人大、市政协监督支持下，市人民政府与全市人民一道，齐心协力，克难攻坚，真抓实干，圆满完成市十一届人大五次会议确定的各项目标任务。

　　（一）发展态势总体良好。发展质量不断提高。完成地区生产总值1619亿元，增长11.4%，总量位居全省第5位。公共财政预算收入108亿元，增长19.4%，总量位居全省第3位，较上年晋升1个位次。社会消费品零售总额540亿元，增长17.1%，增速位居全省第1位。规模以上工业增加值 755亿元，增长16.2 %。金融机构存贷款余额达到 1477亿元、868亿元，分别增长13%和11.1 %。城镇居民人均可支配收入和农民人均纯收入达到20159元和8647 元，分别增长12.1%和14.8%。完成年度节能减排目标任务。发展后劲不断巩固。固定资产投资1380亿元，增长22.2%，总量位居全省第4位，其中工业投资 776亿元，占投资比重的56.2 %。全年实施亿元以上项目613个，增加183个，完成投资902亿元，增长64.7%。天光科技触控光电产业园（一期）、驼人健康产业园等328个亿元以上项目开工建设，河南煤化20万吨乙二醇、百威英博百万吨啤酒生产基地（一期）等306个亿元以上项目竣工投产。省、市重点项目完成年度投资目标。实际利用外资6.4亿美元，利用市外资金420亿元，新签约亿元以上项目201个，中兵北斗二代地面接收机、晋开集团60万吨合成氨80万吨尿素等一批重大项目落户新乡。发展载体不断夯实。新乡经济技术开发区和新乡高新技术开发区同步晋升为国家级园区，平原新区建设步伐加快。8家产业集聚区实现规划调整，合计拓展发展空间42.5平方公里，全市产业集聚区（专业园区）建成区面积超过180平方公里，完成固定资产投资863.5亿元，占全市投资的63%；规模以上工业主营业务收入2346亿元，占全市工业的80%。完成交通基础设施投资23.7亿元，城乡客运一体化全面推进。获批新增建设用地6万余亩，位居全省第2位。发展活力不断显现。通过国家知识产权示范创建城市验收，新培育国家级高新技术企业12家，高远公司“公路养护装备国家工程实验室”获批，“卫华集团院士工作站”成立，新航集团通过国家级企业技术中心认定。专利申请量2651件，发明专利比重达24.9%。成立了服务高校领导小组，校地校企合作深入推进。15家企业被列为省重点上市后备企业，16家企业进入上市实质阶段。发行全省第一只区域集优中小企业集合票据，募集资金2.7亿元。改革不断深入，利用全国农村改革试验区平台，探索推进农村产权制度改革，办理房产证25182本，确定16个集体经济组织产权制度改革试点。以建立土地指标市场为标志的“人地挂钩”政策实施，促进了城乡土地利用一体化建设。国有企业改革稳步推进。文化体制改革顺利完成。启动聚能警务战略。全面实施“四大放心”工程，食品安全综合监管体制机制不断健全。不断完善公共资源交易中心运作模式，进入中心交易项目1649项，增收节支9.4亿元。省管县和经济发达镇体制改革试点有序推进。减少市直和县级事业机构147家。

　　（二）发展方式加快转变。三次产业比重调整到12.4∶ 58.7∶28.9，二、三产业比重达到87.6%，提高0.2个百分点。新型工业化加快发展。六大产业实现主营业务收入2886.5亿元，占全市规模以上工业的97.8%。新兴产业增加值增长24.4%，高于全市规模以上工业8.2个百分点。“车电分离、换电为主”的电动车运营模式获国家有关部门、业界专家和运营商充分认可。“神九”、“蛟龙”、辽宁号航母核心部件均有新乡企业贡献。9家企业被确定为省“两化”融合示范企业。卫华集团荣获省长质量奖，新增1家中国驰名商标，被确定为首批“全国质量强市示范城市”创建城市。现代农业发展水平不断提升。实施高标准粮田建设97万亩，新增有效灌溉面积2万亩，再夺省“红旗渠精神杯”。连续13年实现建设占用耕地“占补平衡”，代表河南省接受国家耕地保护目标考核。粮食单产、总产均创历史最好水平，实现“九连增”，首次荣获“全国粮食生产先进市”称号。现代服务业稳步发展。总投资60亿元的宝龙城市广场等一批大型商贸物流项目建成开业，海宁皮革城成功创建国家3A级景区，关帝庙历史文化街等17个服务业项目纳入省服务业提速计划。第三产业增加值占GDP比重提高0.6个百分点。环境保护不断加强。着力推动以G107和S308绿化为代表的城乡绿化一体化工程，完成林业生态建设11.3万亩，全国绿化模范城市通过验收。5条河流出境断面达标率为91.7%，48个农村环境连片综合整治项目全部完工，饮用水源地取水水质达标率继续保持100%，被命名为首批“河南省宜居城市”。

　　（三）城乡建设步伐加快。实施基础设施、旧城改造、生态环境等8大类728项工程，总投资1015亿元。中心城区品质持续提升。新区核心区150个重点项目全年完成投资60亿元。铁西新城建设提上重要议事日程。25个城中村改造项目完成征收拆迁108万平方米，开工66万平方米、竣工52万平方米。房地产开发完成投资173亿元，增长10.7%。石武高铁新乡站投入使用。赵定河综合治理工程12公里河道开挖基本完成，铺设主园路1094米、次园路1150米。县城内涵式发展步伐加快。持续开展“新城杯”竞赛活动，8 个县（市）完成拆迁310万平方米，房地产开发面积730万平方米。镇（乡）区发展建设全面展开。深入全市121个乡镇调研，出台了加快镇（乡）区开发建设的指导意见和实施方案，推进了100家重点企业和100家房地产开发企业与镇（乡）区发展对接，以20个示范中心镇建设为引领，镇（乡）区在现代城镇体系中的关键节点作用不断强化。新型农村社区稳步推进。各类资金投入98.9亿元，新开工建房面积1171.3万平方米，新入住农户4.3万户，完成旧宅拆迁2.5万亩，复垦或恢复生态1.2万亩。将社区建设与扶贫开发结合，完成52个整村推进和364户、1750人扶贫搬迁任务。城市规划管理进一步加强。基本达到市、县建成区和重点镇主要街区控制性详细规划全覆盖，涵盖城乡的全域规划体系初步构建。市区三（四）轮车整治规范成效显著，以数字城管为支撑的城乡管理一体化平台不断健全，顺利通过国家卫生城市复审。

　　（四）人民生活持续改善。直接用于社保、就业、保障性住房建设等民生支出172亿元，增长19.7%。城镇新增就业10.1万人，农村劳动力转移就业135.8万人次，城镇登记失业率3.84%，被确定为河南省唯一的国家失业预警试点市。实现城乡居民社会养老保险国家试点全覆盖，受到国务院表彰。养老、医疗、工伤、生育、失业保险等均实现扩面提质。建立完善社会救助制度，30余万名城乡低保、农村五保对象、孤儿、优抚对象等得到及时救助。新开工各类保障性住房2.8万套。率先在全省成立环卫爱心基金会救助保障困难环卫工人。小学、初中阶段入学率均达到100％，完成校安工程年度建设目标任务。市区社区卫生服务网络覆盖人口100%，标准化村卫生室建成率100%，新农合参合率99.36%。实现人口统筹发展，优生优育水平不断提高。农家书屋实现行政村全覆盖。在300多个城乡社区和城市公共场所配备健身器材1842套，成功举办第十届运动会。新解决40万农村居民和7.5万农村师生饮水安全问题。新投放公交车200辆，不断探索出租车经营新模式。南水北调新乡段干渠工程及库区移民工作扎实推进。实现全国双拥模范城“五连冠”，驻新部队和单位为新乡的各项事业进步作出了重要贡献。平安建设深入推进，着力构建以法律为主导的群众诉求表达渠道和处理机制，实现全省平安建设先进市“六连冠”，公众安全感指数连续4年位居全省前3位。安全生产工作综合排名全省第一，事故死亡人数低于省控制进度指标84.3个百分点。民族地区生产生活条件不断改善，社会大局保持稳定。一年来，全市上下深入开展作风教育整顿和“三抓两促”活动，工作机制不断健全，作风持续转变，政府自身建设不断加强，人大代表议案、建议和政协委员提案办结率均为100%。统计、审计、外事、侨务、史志、人防、气象、社科研究、防灾减灾等各项事业全面均衡发展。

　　各位代表：2012年是国内外经济形势复杂、面临困难较多的一年，面对经济下行、要素约束、区域竞争以及我市自身改革发展任务繁重等诸多压力和挑战，全市上下行动迅速、措施有力、应对及时，持续加快了发展、巩固了后劲、彰显了亮点。一年来，我们重点从以下方面推进工作落实：一是坚定全面系统地贯彻部署“两不三新”“三化”协调科学发展战略。通过全面系统地打造四级城镇体系，不断强化新型城镇化的引领作用，城镇化率达到44.69%，高于全省2.49个百分点；大力发展新兴产业，打造六大千亿元产业，新型工业化主导作用进一步增强，工业化率达到51.7%；发挥农业科技优势，农业现代化基础地位更加牢固，“三化”协调科学发展的态势日益凸显。二是建立起了比较有效的抓落实的工作推进机制。完善了重大项目周例会、城建项目例会、银企对接月例会、现场观摩会等重点工作推进机制；建立健全了政府组织推动，企业和高校、研究单位联盟为主体，行业协会共同参与的重大产业推进机制；坚持企业服务长效机制，实行部门24小时能够办公的制度，促进了各项工作落实。三是面对严峻经济形势应对及时、措施有力。对全年严峻形势的预判做到见事早、行动快，年初即确定“稳增长、保态势、优结构、惠民生”应对思路；加强对财税收入、用电量等先行指标的监测和研判，每季度召开经济运行会议，有针对性地采取应对措施；对省委、省政府作出的决策部署迅速落实，及时对接，并确立我市的工作重点，建立台账，逐项推进，促进了经济运行由缓中趋稳向筑底企稳转变。四是敢于创新，不断破解发展中的难题。始终把创新作为破解难题、推动工作的动力，在产业关键核心技术突破、土地和资金等瓶颈制约破解、各项改革试验试点探索等方面都取得了新的成绩，进一步释放了发展的活力。五是齐心协力、团结协作，努力形成工作合力。全市上下干事创业氛围浓厚，在争取上级支持、争取重大项目落地等工作中，各级、各部门咬定青山不放松，守之不失、始终不渝，团结协作、合力攻坚，展示出了良好的精神风貌。

　　各位代表：一年来工作成绩的取得，是省委、省政府正确领导的结果，是市四大班子团结拼搏的结果，是全市人民共同努力的结果，是方方面面大力支持的结果。在此，我代表市人民政府，向所有关心、支持和参与新乡发展建设的同志们、朋友们表示衷心的感谢，并致以崇高的敬意！

　　各位代表：在肯定成绩的同时，我们清醒地认识到经济社会发展中还存在不少矛盾和问题：一是保态势的压力较大。尽管我市经济正在逐步回升，但宏观经济环境的不确定因素依然较多，稳定增长的基础并不牢固，同时主要指标的人均值偏低，区域竞争更加激烈，晋位升级的压力较大。二是增后劲的压力较大。各县（市）、区重大项目建设进展不平衡，重大支撑项目有待持续跟进，集群式招商等工作力度有待进一步加强；支撑产业有待做优做强，金融和资本市场利用有待拓宽，各项改革试验试点有待细化深入，创新驱动、内生增长的机制尚未形成。三是促协调的任务较重。农业的巩固、县域的协调、城乡的统筹、生态的共进等都是我们面临的重大课题；特别是随着人民群众对公共服务需求的不断上升，民生和社会事业领域的投入与发展还显不足。四是转作风的任务较重。政府系统抓落实的力度需要加强，服务水平需要提升，科学管用的工作机制需要不断探索完善；干事的精力不够集中，廉洁自律的意识不够强，为民服务的意识不够强，群众路线坚持不够好、作风不够扎实，有的问题还相当严重。这些都需要在今后的工作中高度重视，并切实认真加以改进解决。

　　二、2013年工作重点

　　2013年是全面贯彻落实党的十八大精神、推进《中原经济区规划》的开局年，是实现我市晋位升级目标的攻坚年。面对新形势、新任务，既要增强忧患意识，更要坚定信心，变压力为动力，化挑战为机遇，持续求进。做好今年工作，要围绕“一个总目标、两条主线”要求，以提高经济增长质量和效益为中心，坚持走“两不三新”“三化”协调科学发展路子，推动工业化、信息化、城镇化、农业现代化融合同步发展，全力以赴稳增长、保态势，抓创新、促转型，惠民生、促和谐，推动中原经济区强市建设取得新突破，为全面建成幸福新乡小康社会打下坚实基础。

　　今年经济社会预期目标是：生产总值增长10.3%，其中一、二、三次产业分别增长3.8%、13.3%和7%；全社会固定资产投资增长21%；社会消费品零售总额增长16%；公共财政预算收入增长13%；城镇居民人均可支配收入、农民人均纯收入均增长9.5%；城镇登记失业率控制在4.5%以内；人口自然增长率控制在6.5‰以内；居民消费价格指数涨幅3.5%左右；节能减排指标控制在省定目标之内。同时，按照党的十八大提出的到2020年全面建成小康社会要求，综合考虑我市各方面条件，市委、市政府提出新乡全面建成小康社会的三个具体目标：一是提前一年全面建成小康社会。二是实现生产总值和城乡居民人均收入在2012年的基础上翻一番以上。三是人均生产总值、城镇居民人均可支配收入、农民人均纯收入达到全国平均水平。实现上述目标，对全市工作提出了更高要求，我们要牢牢把握发展这个第一要务，以更大的力度、更高的标准，全力抓好各项工作落实。

　　 今年要重点推进以下8个方面的工作：

　　（一）强力提升新型城镇化引领作用。优化城镇体系布局。树立全域新乡理念，优化城镇空间形态格局、产业布局、交通布局、生态布局，推动城市发展由速度型向品质提升型转变。按照“一主、两副、五组团”要求，统筹推进中心城市、县城、镇区和新型农村社区四级城镇体系建设，构建放射状、网络化、开放型布局，建设现代田园城市。坚持有序推进农业转移人口市民化，坚持现有市民权益的维护和改善，坚持数量质量并重和科学生态城镇化，加快构建新型现代城镇体系，实现城镇化率提高1.8个百分点。打造高品质城市核心区。优化城市140平方公里核心城区的空间布局和功能分区。加快东部新区建设，完善城市功能，大力推进高铁车站及周边区域、平原文化艺术中心、平原体育会展中心、银河科技城、医疗健康中心、东区医院等项目建设，完善核心区城市功能，打造中心城区第二商业板块、创新板块、体育运动板块和健康板块。以凤凰山森林公园区域为依托，支持北部休闲区建设。完成商务中心区和特色商业区规划编制。加快旧城有机更新、均衡发展，启动23个城中村改造项目，完成拆迁120万平方米，新开工建设100万平方米；开工建设棚户区改造项目11个，完成拆迁44万平方米，建设安置房4800套。搭建融资平台，做好项目谋划推进，全面启动铁西新城规划建设。加快推进军用直升机场搬迁。提升基础设施保障能力，统筹城市道路及各类管网建设，开工建设平原路缓堵保畅工程、石武高铁站前广场地下交通枢纽工程等重点项目。按照南环东段标准，加快实施南环西段、西环路提升改造，加快推进中原东路东延、平原路东延等工程建设。实施部分路口道路渠化、左行待转改造，建设公交港湾。建设高品质城市（县城）组团。强力推进平原新区建设，打造高品质城市新区，构建经济发展的重要增长点。持续开展“新城杯”竞赛，以拆迁引领县城内涵式发展。8个县（市）完成拆迁330万平方米，其中辉县市不低于60万平方米，卫辉市、长垣县不低于50万平方米，新乡县、原阳县不低于40万平方米，获嘉县、延津县、封丘县不低于30万平方米。加快完善基础和公共服务设施，各城市组团至少要培育一个中心商务功能区或特色商业区。辉县市、长垣县力争5年内率先发展成为人口集聚能力强、功能完善、人口30万左右的中等城市；卫辉市、获嘉县、原阳县、延津县、封丘县发展成为各具特色、功能完善、人口接近20万的宜居小城市。全面推进、重点突破镇（乡）区发展建设。实施中心镇功能提升工程，自2013年起，市级财政连续5年每年安排2000万元专项建设扶持资金，支持镇（乡）区基础设施和公共服务设施建设，提高承接中心城市、县城辐射和带动农村发展能力。把镇（乡）区发展建设与新型农村社区、产业集聚区规划建设相结合，坚持用现代城市理念规划建设镇（乡）区，通过引进高水平有实力的开发商，加快基础设施和公共服务设施建设，尽快形成“井”字型或网状城镇框架。20个示范中心镇和48个重点乡镇，要强力推进镇区开发、产业集聚区、周边新型农村社区建设，逐步发展成为具有一定规模的小城市。实施扩权强镇，建立结构合理、管理科学、运转高效、依法规范的管理体制。科学有序、积极推进新型农村社区建设。在完善提升已基本建成的15个示范社区的基础上，重点抓好200个重点社区建设，推进其他已启动社区建设，全年实现新开工建房1200万平方米，新入住农户4万户，37个示范社区基本建成。落实旧宅拆迁利益补偿机制，在引入市场机制上进行探索，新拆除旧宅2.5万亩、复垦1.5万亩。继续实施农民增收致富工程，加快推进27个农民创业园建设，促进农民就近就业。强化规划引领和交通支撑。完成全域新乡现代城镇体系规划编制深化，进行全市的控规梳理，完成121个镇（乡）区总体规划和控制性详细规划并通过评审。深入实施“无线城市”和“智慧新乡”建设。大力推进数字城管城乡一体化，开展户外广告和门店招牌规范整治，再创全省城市精细化管理优秀城市。加快推进郑州至新乡城际铁路前期工作，启动郑焦高速东延原阳至大广高速、焦作至辉县至林州高速前期工作，全力实施新晋高速、鹤辉高速和长济高速东明黄河大桥等重点项目建设，抓好G107马村立交至黄河大堤续建、S230薄口线薄壁至G107、S226翟阳线卫辉市至新乡经济技术开发区等项目建设，加快推进城乡客运一体化，完成1个~2个县（市）线路改造任务。

　　（二）打造有持续竞争力的现代产业体系。实施六大千亿元产业板块培育工程。新型电池与电动车方面，大力发展动力及储能电池产业集群、新能源汽车产业集群以及专用汽车基地。启动“中华牌”电池腾飞方案。谋划在我市召开全国性的电池产业发展论坛暨电池产品产销对接会。加强与福汽集团、神华集团的对接合作。生物与新医药方面，完善生物技术药物、化学成品药、现代中药、卫材和医疗器械4条主导产业链，抓好华兰生物与丹麦CMC公司合作、双鹭生物医药产业园等项目实施。现代煤化工方面，围绕22个产业链项目，以甲醇、乙二醇、烯烃为基础，重点发展聚酯、汽车防冻液等乙二醇下游延伸产品。制冷及电子信息产业方面，快速壮大制冷终端产业规模，带动配套产业和制冷材料产业发展，打造中原经济区最大的家电制造和配套基地。推进新飞、中航两大制冷企业做大做强，打造全国重要的冷藏车生产基地。加强与浙江博克电器集团的对接与合作，做好压缩机项目引进，努力提高制冷产业本地配套率。发展壮大信息制造业，争取中兵北斗二代地面接收机项目开工建设，抓好中兵通信科技产业园、中国电波科技城、天光科技触控光电产业园等项目的实施。特色装备制造方面，支持起重、振动、数控、铸造等优势产品做大做强，打造中原经济区特色装备制造基地；推进卫华集团重型装备产业园、豫飞重工船用集装箱起重机等项目实施。传统优势产业方面，用先进适用技术改造提升食品、纺织、造纸、建材等传统产业，提升产业竞争力，推进河南新百维食品公司年产万吨食品添加剂、新亚集团40万吨食品卡纸等项目建设。千方百计壮大实体经济。坚持外引与内培并重，扶持本地企业做大做强，支持中小微企业壮大。大力实施产量产能扩增计划，分行业、分专题开展产销对接、产业链对接，帮助企业开拓市场。深入开展企业服务活动，加大重点企业和行业问题解决力度。加快推进省级两化融合试验区和企业综合信息服务平台建设，不断提升信息化水平。强力推进产业集聚区建设。将2013年确定为产业集聚区招商引资工作年，重心从基础设施向集群招商转移，省级产业集聚区完成招商引资额增长40%，固定资产投资700亿元，主营业务收入1500亿元。强化学校、医院、商业等功能集合构建。支持新乡高新技术开发区在高新技术产业、新乡经济技术开发区在先进制造业等方面加快发展。严格土地开发利用管理，大力推进节约集约用地，不断提高土地综合利用率和收益率。

　　（三）大力推动新型农业现代化取得新突破。深入贯彻中央一号文件精神，认真落实各项强农惠农富农政策，加快构建新型农业经营体系和现代农业产业体系，努力提高农业综合生产能力。千方百计抓好粮食生产。积极实施高标准粮田 “百千万工程”， 建设高标准粮田70万亩，新增有效灌溉面积2万亩，全面完成南水北调渠首及沿线土地整治重大项目第三年度目标任务，增强农业综合生产能力，粮食产量稳定在40亿公斤以上，打造河南粮食生产核心区优质高产高效示范区。大力实施现代农业产业化集群培育工程。积极培育优质小麦、大米等11类农业产业化集群，其中销售收入10亿元以上的集群3个，新增市级以上农产品品牌5个。以茅台集团、酒鬼酒等小麦原料基地建设为契机，大力发展订单农业，组建完善小麦产业集团，谋划中国小麦产业博览会，打造中国第一麦、中国第一米及鲜切花等品牌。大力发展高效经济作物，种植面积稳定在350万亩以上。加快雏鹰农牧集团生猪产业化基地等项目建设，培育一批优势明显的大型畜牧养殖加工企业。新发展农民专业合作社200家，辐射带动农户达到35万户以上。提升农业科技支撑能力。推进现代农业技术研发和成果转化示范中心建设，依托中国农科院组织申报新乡县国家级现代农业示范区，深化院地对接，加快科技成果转化。大力发展品牌农业、高效农业、生态农业和高端农业，促进农业增效、农民增收。

　　（四）坚定不移地实施引资项目双带动战略。提升招商引资的针对性和有效性。创新优化招商引资方式，围绕电池与电动车、制冷及电子信息、现代煤化工等产业链条和图谱梳理出的135个产业链弱项和缺项及160个重点项目，有针对性地开展补链、延链和集群式招商，抓好赴珠三角地区的电池、赴长三角地区的制冷和汽车及零部件等专业招商会，全力跟踪天光科技触控光电产业园、中兵集团特种汽车、上塑集团新型系列塑料管道生产、娃哈哈集团50万吨果葡糖浆、德国KME集团合金铜板带及上海松芝轨道车辆空调、北京地铁车辆装备等重大产业集群和产业项目，争取一批落地实施。实际利用外资和市外资金分别增长10%和15%，实现集群式招商100亿元，引资总量保持全省前列。加大综合保税区申建工作力度，加快新乡出入境检验检疫局建设，完成出口9.05亿美元，增长15%。强力推进重点项目建设。实施1200个5000万元以上项目，新开工627个。其中，省重点项目110个，市重点项目300个，完成投资1000亿元以上。重点抓好中航工业新乡航空高科技园二期、新能源电动汽车生产基地、胜华电缆特种电缆产业园、中原经济区大学科技城等156个5亿元以上项目。力争渠东电厂2×30万千瓦热电联产机组、辉县汽配欧玛汽车部件工业园、双鹭阿德福韦酯等原料药及制剂等重大项目竣工投产。健全重大项目动态储备库，再谋划一批投资规模大、科技含量高、产业链条长、带动作用强的重大项目，加快推进赵固综合利用电厂2×30万千瓦热电联产机组、城际铁路云台山支线等项目的前期工作，力争早日开工建设。深入贯彻推进省“33155”行动计划17项专项工作方案，做好政策、项目、资金争取工作。

　　（五）持续推进现代服务业发展壮大。大力发展金融服务业。实施企业上市引导计划，争取金龙铜管、天丰节能板材、日升数控年内发行上市，推动拓新生化等企业进入辅导，加强上市后备企业培育。做好投资集团9亿元企业债、心连心化肥3亿元短期融资券前期工作，推广区域集优债务融资模式，实施小巨人信贷培育计划，推动兴业银行、中信银行在我市设立分支机构，争取设立1家~2家村镇银行，新增贷款90亿元以上。建立市应急转贷资金，防范和化解金融风险。大力发展旅游业。强力推进南太行旅游资源整合，完成南太行旅游规划编制和旅游集团公司组建，实施旅游环路、集散服务中心等重点项目建设，力争创建成国家5A级景区。着力打造南太行山水游和体育攀岩、牧野文化游、黄河文化及沿黄生态游、南水北调生态文化游等精品线路。抢抓高铁旅游商机，挖掘各种旅游资源，打造丰富多样、独具特色的旅游产品。国内旅游收入、接待国内游客人数分别增长15%和10%。大力发展商贸物流业。加快推进香港豪德集团商贸物流园、香港宏安集团蔬菜物流园等重大商贸物流项目建设，提升餐饮等传统服务业发展水平。抓好万村千乡市场工程、新网工程、农产品流通体系等民生工程实施，积极培育现代城市服务业态，建设区域性商贸中心城市。

　　（六）深入实施创新驱动发展战略。加强创新平台建设。坚持把突破和掌握核心技术作为产业发展的关键，组织实施科隆集团“动力锂离子电池正极材料研发”等10项~15项重大产业科技攻关项目。力争河南（新乡）电池研究院建成国家级工程技术中心，抓好新乡医学院干细胞与生物治疗技术研究中心、华兰生物研发和中试中心、高远公司公路养护装备国家工程实验室建设。组织申报省级工程技术研究中心3家，新建市级工程技术研究中心、重点实验室20家。提升企业自主创新能力。在生物医药、煤化工、新型电池等领域实施重大科技专项，突破10项产业关键核心技术。组织申报省级以上科技项目30项，提升国家知识产权示范城市创建水平，专利申报量保持在2000件以上，其中发明专利占20%以上。争创中国驰名商标2个、省著名商标20个。加强政产学研结合。完善校市合作机制，利用高校科技优势加强协同创新，促进高校科研成果就地转化。支持高新技术产业开发区和新乡经济技术开发区围绕战略新兴产业建设专业化科技企业孵化器。推动生物医药等领域的产业技术创新战略联盟建设。深化各项改革。围绕全国农村改革试验区建设，深入推进城乡一体化制度创新，抓好农村“两权一房”抵（质）押贷款工作和农村集体产权制度改革，推进长垣县农村土地流转试点。深化土地管理制度改革创新，完善人地挂钩试点政策体系。稳妥推进市属国有企业改革，处理好国有企业改制遗留问题，有序推进国有企业厂办大集体企业改革，推进市直部门所属企业脱钩工作。深化工资分配制度改革。探索试点县级公立医院改革。构建信息化条件下全新警务工作运行机制。

　　（七）加快推进美丽新乡建设。全力打造市民亲水空间。以“渠相连、水常清、水常流”为目标，完成赵定河综合治理一期工程和主要河段景观建设；抓住大运河新乡段申遗机遇，谋划推进卫河市区段生态文化建设；完成南护市沟开挖疏浚，实现市区人民胜利渠、西孟姜女河和卫河的有机贯通。抓好南水北调受水配套工程实施；稳步推进引黄调蓄和引黄补源工程，加强雨水收集利用，加大地下水资源保护力度。推进水环境综合治理，5个省控地表水责任目标断面水质达标率达到省政府要求，城市集中饮用水源地取水水质达标率98%以上。推进城乡绿化一体化。加快高铁站生态湖公园等重大绿化项目建设，实施道路、游园、河渠等绿化工程56项，城市绿化覆盖率达到41.3%以上，打造三季有花、四季常青的城市绿化体系。全面完善提高廊道绿化水平，推进城市组团之间生态化隔离，提高县城绿化建设水平，完善平原农区林网，完成林业生态建设工程10万亩。强化节能减排和环境保护。积极推进全国环保模范城创建，严格执行环境影响评价制度，从源头控制新增污染。建立大气污染防治联防联控机制，针对雾霾天气，抓好机动车、城市燃煤锅炉、道路扬尘等领域的大气污染专项整治，按照省工作部署和时间节点安排，尽早开展PM2.5大气环境监测工作，市区大气环境质量优良天数比例达到80%以上。按期完成辉县市、获嘉县、封丘县和市区骆驼湾污水处理厂升级改造任务，加快贾屯、平原新区等新建污水处理厂建设进程，推进电力企业和水泥企业脱硝设施建设。加强农村环境保护，实现30%的规模化畜禽饲养场配套建设污染防治设施。强化重点区域环境整治，严厉打击环境违法行为。深入实施全民素质提升工程。不断完善精神文明建设长效机制，广泛开展“爱我家园、共建文明幸福新乡”活动，推动城市文明程度和市民文明素质不断提高。大力弘扬新乡英模群体精神，抓好未成年人思想道德建设。全面落实“六五”普法规划，增强全社会法制观念，完善人民调解、行政调解、司法调解“三位一体”的“大调解”体系。大力发展文化事业，抓好市图书馆二期工程、市群众艺术馆新馆建设，广泛开展群众文化活动。支持驻新部队建设，坚持拥在平时、拥在关键、拥在最需要的时候，争创省级双拥模范城“七连冠”。

　　（八）全力加大民生工程实施力度。大力发展教育事业。做好市县两级教育发展专项规划，完善市、县、乡、村四级教育服务体系。加快发展学前教育，均衡发展义务教育，全面推进素质教育，着力提高基础教育教学质量，扩大优质教育规模。改造农村义务教育薄弱学校，完成一中东校区建设，开工建设育才小学东校区。深化职教改革创新，启动职业技术学院新校区二期规划建设，加快新乡幼师建设步伐。支持新联学院、新科学院发展，加快三全学院、河南财经学院平原新区校区建设。健全完善安保制度和突发事件应急处置机制，维护校园安全。千方百计增加就业。深入开展创业型城市创建，城镇新增就业7.3万人，下岗失业人员再就业2.25万人，就业困难对象再就业0.72万人，农村劳动力转移就业136.5万人次。建立完善工资集体协商共决机制、正常增长机制和支付保障机制。常态开展进城务工人员工资拖欠整治行动，构建和谐劳动关系。健全社会保障体系。努力扩大社会保险覆盖范围，城镇职工基本养老保险人数达到77.6万人，城乡居民社会养老保险参保人数达到253万人，失业保险参保总数保持在44万人，城镇基本医疗保险、工伤保险、生育保险参保人数分别达到141.5万人、52.3万人和29.7万人，新农合参保率保持99%以上。继续完善城乡居民社会养老保险工作机制，进一步扩大覆盖范围。加强和改进最低生活保障工作，推进社会救助规范化。积极应对老龄化问题，抓好老龄组织、社会养老服务体系建设和居家养老服务工作。新开工各类保障性住房3.4万套。争取成为住房公积金贷款支持保障性住房建设试点城市。努力改善群众医疗条件。加快推进新区三级医院、中心医院门诊楼、市一院全科医生培训大楼、新乡卫校新址建设，实施市妇幼保健院和市结核病防治所建设工作，加大12个卫生监督所、45个乡镇卫生院建设力度。为困难群众减免医疗费用1000万元以上，为1500名贫困白内障患者免费实施手术，做好国家卫生部“健康快车”再次停靠新乡工作。强化食品药品安全监管。严格落实政府属地管理和部门监管责任，健全食品安全监管体系和工作机制，完善食品安全信用制度，扩大食品安全风险监测覆盖面，加大对乳制品、肉制品、蔬菜、食用油以及食品生产、加工、餐饮经营等重点产品和环节的整治力度；深入开展药品安全专项整治，严厉打击制售假劣药品违法犯罪行为，维护人民群众饮食用药安全。办好其他社会事业。再解决20万人的饮水安全问题。新改造农村公路200公里。对46个贫困村实施整村推进，完成260户、1200人深石山区独居散居贫困农户搬迁扶贫任务。继续做好市区公共体育便民设施配备。深入开展爱国卫生运动，巩固国家卫生城市创建成果。稳定适度低生育水平，加强计生服务能力建设，提高出生人口素质。发挥工会、共青团、妇联等人民团体和社会组织在社会管理中的重要作用。深入开展质量立市活动，创建全国质量强市示范城市。抓好安全生产，努力实现事故死亡人数下降。扎实推进平安新乡建设，再创“全国社会治安综合治理优秀市”，夺取“长安杯”。深入开展民族团结进步和宗教工作创建活动，扶持少数民族聚居地区经济发展，促进民族团结进步和宗教和谐稳定。加强和创新社会管理，抓好基层基础工作，解决群众合理诉求，妥善处置突发事件。增加灾害救助投入，提高自然灾害应对能力。完善预防预警和应急处置体系，维护社会和谐稳定。做好新闻出版、广播电视、统计、史志、外事、侨务、档案、人防等工作，促进各项社会事业协调发展。

　　三、加强政府自身建设

　　全面贯彻党的十八大精神，认真落实中央、省委和市委关于改进工作作风、密切联系群众的各项要求，坚持“三具两基一抓手”，推进“两转两提”，深入开展以“抓具体、抓规范、抓创新，促转变、促提升”为主的“三抓两促”活动，不断提高行政管理和服务水平，为实现经济持续快速健康发展和社会和谐稳定提供坚强保障。

　　（一）坚持以人为本，执政为民。牢固树立宗旨意识，进一步增强群众观念。“不患无策，而患无心”。要树立执政为民之心，人民利益至上之心，学习群众、贴近群众、引导教育群众之心，使人民至上、执政为民的理念内化于心、外化于行。要自觉贯彻党的群众路线，建立完善简单实用直接联系群众的制度，完善群众意见诉求表达反馈和答复的机制，充分保障群众的知情权、参与权、选择权和监督权。要以身作则、率先垂范，带头转变工作作风，认真精简会议活动、文件简报，减少检查评比、迎来送往，正文风、转会风、清政风，到矛盾集中、群众意见多的地方研究解决问题，不慕虚名，把发展问题一个一个解决好，把民生事情一件一件办好。

　　（二）坚持依法行政，认真履责。要切实增强依法决策、依法行政的意识和能力，建立完善重大决策调查研究、征求意见、风险评估及听证、公示等制度，严格按法定职责和程序行政，按规章制度办事。自觉接受人大及其常委会的依法监督和政协的民主监督，抓好人大代表议案、建议和政协委员提案办理。认真听取各民主党派、工商联、无党派人士及各人民团体的意见，广泛接受社会公众和新闻舆论监督。严格行政执法责任制，完善行政执法监督制约机制。要按照“于法周严、于事简便”的原则，把遵循规则作为一种习惯，做到严格执行、依法行政。

　　（三）坚持解放思想，勇于创新。中华民族，生生不息，其命维新。新乡之新，在于常新，创新是我们事业发展的不竭动力。要深入开展“三抓两促”，突出创新主题，实施“创新驱动发展年”活动，跳出旧思维，想出新办法，拿出新举措，以创新的意识、创新的胆略、创新的思维来谋划推进各项工作，努力在科技、管理、机制、环境等方面取得更大突破。要把创新贯穿政府工作的始终，革除与发展要求不相适应的思维定势和工作方法，在制定政策、提出规划、组织实施、督促检查等各环节努力探索科学有效、简单管用的机制，破解发展难题、扫除发展障碍。

　　（四）坚持一线作为，提高效能。空谈误国，实干兴邦。必须把干事创业作为政府工作的核心价值，将重点工作落实到具体项目和人员，做到问题在一线解决、工作在一线落实。推进政府绩效管理，强化执行力建设，加大问责追究力度，加大查处行政不作为、乱作为、庸散懒软等问题的力度，构建监督到位、考核到人的推进机制，确保政令畅通、工作落实，树立“信念坚定、忠诚履职、直面矛盾、务实善干、一抓到底、运转高效”的队伍形象。

　　（五）坚持清廉务实，勤俭节约。要认真落实党风廉政建设责任制和领导干部廉洁自律规定，深入推进“四位一体”服务监督体系建设，健全行政监察、审计监督等内部监督机制，做到关口前移，拓展从源头上防治腐败的领域。要全面贯彻中央关于改进工作作风、密切联系群众的八项规定，领导干部带头落实，大力弘扬艰苦奋斗、勤俭节约的作风，严格预算支出管理，开展绩效预算试点，坚决制止铺张浪费和奢侈享乐行为，建设职能科学、结构优化、廉洁高效、人民满意的服务型政府。

　　各位代表！新乡发展充满希望，新乡人民充满期待。让我们紧密团结在以习近平同志为总书记的党中央周围，在省委、省政府和市委的坚强领导下，全面贯彻落实党的十八大精神，坚定信心、团结拼搏、携手共进，奋力谱写富民强市幸福新乡新篇章！
